# Accelerating genomic workflows using NVIDIA Parabricks

**DOI:** 10.1186/s12859-023-05292-2

**Published:** 2023-05-31

**Authors:** Kyle A. O’Connell, Zelaikha B. Yosufzai, Ross A. Campbell, Collin J. Lobb, Haley T. Engelken, Laura M. Gorrell, Thad B. Carlson, Josh J. Catana, Dina Mikdadi, Vivien R. Bonazzi, Juergen A. Klenk

**Affiliations:** 1 Health Data and AI, Deloitte Consulting LLP, VA 22009 Arlington, USA; 2 Cloud Managed Services, Deloitte Consulting LLP, Detroit, MI 48226 USA

**Keywords:** GPU acceleration, NVIDIA Parabricks, Cloud computing, Amazon Web Services, Google Cloud Platform

## Abstract

**Background:**

As genome sequencing becomes better integrated into scientific research, government policy, and personalized medicine, the primary challenge for researchers is shifting from generating raw data to analyzing these vast datasets. Although much work has been done to reduce compute times using various configurations of traditional CPU computing infrastructures, Graphics Processing Units (GPUs) offer opportunities to accelerate genomic workflows by orders of magnitude. Here we benchmark one GPU-accelerated software suite called NVIDIA Parabricks on Amazon Web Services (AWS), Google Cloud Platform (GCP), and an NVIDIA DGX cluster. We benchmarked six variant calling pipelines, including two germline callers (HaplotypeCaller and DeepVariant) and four somatic callers (Mutect2, Muse, LoFreq, SomaticSniper).

**Results:**

We achieved up to 65 × acceleration with germline variant callers, bringing HaplotypeCaller runtimes down from 36 h to 33 min on AWS, 35 min on GCP, and 24 min on the NVIDIA DGX. Somatic callers exhibited more variation between the number of GPUs and computing platforms. On cloud platforms, GPU-accelerated germline callers resulted in cost savings compared with CPU runs, whereas some somatic callers were more expensive than CPU runs because their GPU acceleration was not sufficient to overcome the increased GPU cost.

**Conclusions:**

Germline variant callers scaled well with the number of GPUs across platforms, whereas somatic variant callers exhibited more variation in the number of GPUs with the fastest runtimes, suggesting that, at least with the version of Parabricks used here, these workflows are less GPU optimized and require benchmarking on the platform of choice before being deployed at production scales. Our study demonstrates that GPUs can be used to greatly accelerate genomic workflows, thus bringing closer to grasp urgent societal advances in the areas of biosurveillance and personalized medicine.

**Supplementary Information:**

The online version contains supplementary material available at 10.1186/s12859-023-05292-2.

## Background

As the cost of genome sequencing continues to decrease, genomic datasets grow in both size and availability [1]. These processes will greatly enhance aims such as whole genome biosurveillance and personalized medicine [2, 3]. However, one challenge to attaining these goals is the computational burden of analyzing large amounts of genomic sequence data [4]. Two trends (among others) are helping to ameliorate this burden. The first is the migration to Cloud for data analysis and storage, and the second is the use of Graphics Processing Units (GPUs) to accelerate data processing and analysis [5, 6]. We discuss each of these trends in this article.

Cloud computing addresses many of the challenges associated with large whole genome sequencing projects, which can suffer from siloed data, long download times, and slow workflow runtimes [7]. Several papers have reviewed the potential of cloud platforms for sequence data storage, sharing, and analysis [1, 5, 8–12], thus here we focus on one cloud computing challenge, how to select the right compute configuration to optimize cost and performance [13, 14].

GPU acceleration in either a cloud or High Performance Computing (HPC) environment makes rapid genomic analysis possible at previously unattainable scales. While these are still early days for GPU-acceleration in the ‘omics fields, several studies have begun benchmarking various algorithmic and hardware configurations to find the ‘Goldilocks zone’ between cost and performance. Two recent studies [6, 15] benchmarked GATK HaplotypeCaller using the original CPU algorithm and the GPU-accelerated version from NVIDIA Clara™ Parabricks (hereafter Parabricks) on HPC platforms and found notable acceleration (8 × and 21 × speedups respectively) when using GPUs. They also inferred high concordance of SNP calls (~ 99.5%) between the CPU and GPU algorithms suggesting no to low loss of accuracy with the GPU-configured algorithms, for both germline and somatic variant callers [16], a finding also corroborated by [17]. Likewise [18], introduced a new GPU-accelerated pipeline called BaseNumber, which achieved runtimes slightly faster than previous benchmarks using Parabricks.

While the aforementioned studies conducted benchmarking using on-premises computing clusters, only a few studies have begun benchmarking GPU-accelerated algorithms in the cloud. The Parabricks team at NVIDIA benchmarked GATK HaplotypeCaller using Parabricks on Amazon Web Services (AWS) and achieved runtimes as low as 28 min for a 30 × genome with eight A100 NVIDIA GPUs [17]. NVIDIA compared an m5-family virtual machine (32 CPUs, 128 GB Memory; Intel Skylake 8175M or Cascade Lake 8259CL) with several GPU configurations, including g4dn.12xlarge (four T4 GPUs, 48 2.5 GHz Cascade Lake 24C processors), the g4dn.metal (eight T4 GPUs, 96 2.5 GHz Cascade Lake 24C processors, p3dn.24xlarge (8 V100 GPUs, 96 Intel Skylake 8175 CPU processors) and the p4d.24xlarge (8 A100 GPUs, 96 Intel Cascade Lake P-8275CL processors), with the largest acceleration observed with the p4 machine family (with NVIDIA A100s). NVIDIA also benchmarked four somatic callers, and achieved speedups ranging from 4× to 42× with a 50 × human genome. In this somatic variant calling study, they comparing an m5 machine to the g4dn.12xlarge (with four T4 GPUs), though they did not benchmark the newer compute-optimized A100 and V100 GPU machines [16]. Relatedly [13], benchmarked GWAS workflows using Spark Clusters (not NVIDIA Parabricks) on Google Cloud Platform (GCP; using standard n2 machines) and AWS (machines not specified) and found comparable performance between cloud platforms. While several of these studies have shed light on the performance of GATK HaplotypeCaller using Parabricks, fewer studies have compared CPU and GPU performance across a range of germline and somatic variant callers, or compared performance across AWS, GCP and an NVIDIA DGX cluster. Benchmarking a range of algorithms on several platforms and hardware configurations is important to inform future decisions around algorithmic, hardware and platform selection.

Here, we benchmark two germline variant callers and four somatic variant callers comparing traditional × 86 CPU algorithms with GPU-accelerated algorithms implemented with NVIDIA Parabricks on AWS and GCP, and benchmark GPU-accelerated algorithms on an NVIDIA DGX cluster. In the case of GPU-accelerated algorithms, we compare 2, 4, and 8 GPU configurations. For germline callers, we observed speedups of up to 65x (GATK HaplotypeCaller) and found that performance scaled linearly with the number of GPUs. We also found that because GPUs run so quickly, researchers can save money by using them for germline variant callers. Alternatively, somatic variant callers achieved speedups up to 56.8 × for the Mutect2 algorithm, but surprisingly, did not scale linearly with the number of GPUs in some contexts, emphasizing the need for algorithmic benchmarking before embarking on large-scale projects where sub-optimal optimization can substantially increase costs.

## Results

### CPU baseline across cloud platforms

**Fig. 1:**
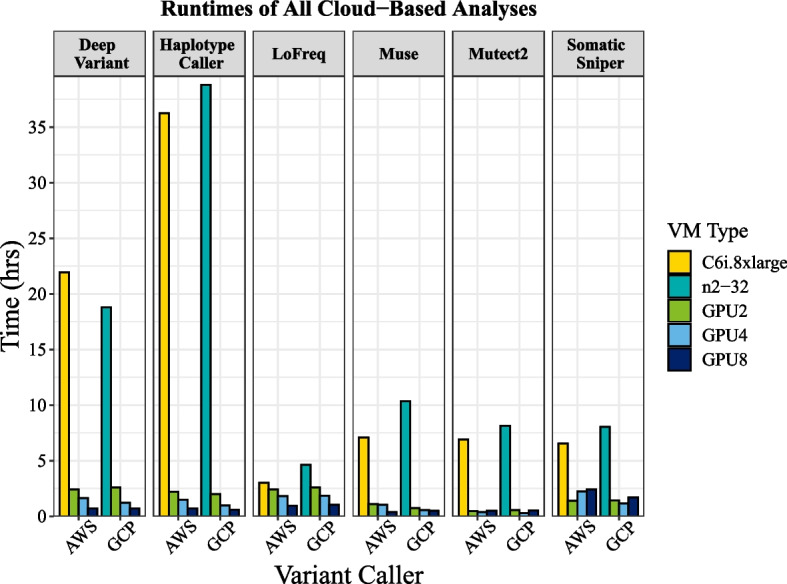
Comparison of execution times of variant calling algorithms on CPU and GPU environments between AWS and GCP. A 32 vCPU machine with the latest processors was used for CPU benchmarking on both cloud platforms. Here we show results for varying numbers of NVIDIA Tesla V100 GPUs running the Parabricks bioinformatics suite for AWS, and NVIDIA Tesla A100 GPUs for GCP

CPU machine performance varied considerably between the c6i machine on AWS (c6i) compared with the n2 machine on GCP for most analyses. For germline analyses, GCP performed faster for DeepVariant (18.8 h) compared with AWS (22 h), whereas AWS performed faster for HaplotypeCaller (36.2 h) compared with GCP (38.8 h; Table 1, Fig. 1). Somatic runtimes favored AWS machines, except for Mutect2, where the n2 machine on GCP ran in 8.1 h compared with 16.9 h on AWS (Table 1, Fig. 1).

**Table 1:** Results of benchmarking for AWS, GCP and NVIDIA DGX workflow runs

Platform	Pipeline	VM-type	Variant-caller	Time (min)	Time (h)	Cost ($)	Fold acceleration	% cost-savings
AWS	Germline	C6i.8xlarge	DeepVariant	1317.3	21.96	29.9	–	–
		GPU2		145.16	2.42	29.61	9.07	0.83
		GPU4		97.07	1.62	19.80	13.57	33.68
		GPU8		42.19	0.7	21.95	31.22	26.49
GCP		n2-32		1128	18.8	32.9	–	–
		GPU2		156	2.6	19.4	7.2	41.03
		GPU4		72	1.2	18.3	15.7	44.38
		GPU8		42.6	0.71	20.9	26.5	36.47
DGX		GPU2		87.9	1.47	–	–	–
		GPU4		49.1	0.82	–	–	–
		GPU8		27.05	0.45	–	–	–
AWS	Germline	C6i.8xlarge	HaplotypeCaller	2175.9	36.26	49.32	–	–
		GPU2		131.99	2.2	26.93	16.49	45.41
		GPU4		88.27	1.47	18	24.65	63.49
		GPU8		41.51	0.69	21.60	52.42	56.21
GCP		n2-32		2328	38.8	67.9	–	–
		GPU2		118.8	1.98	13.5	19.6	80.12
		GPU4		57.6	0.96	14.1	40	79.23
		GPU8		35.4	0.59	17.5	65.8	74.23
DGX		GPU2		64.6	1.08	–	–	–
		GPU4		39	0.65	–	–	–
		GPU8		24.4	0.41	–	–	–
AWS	Somatic	C6i.8xlarge	LoFreq	180.2	3	4.1	–	–
		GPU2		145.14	2.42	29.61	1.24	− 625.07
		GPU4		109.23	1.82	22.28	1.65	− 445.68
		GPU8		57.18	0.95	29.75	3.15	− 628.55
GCP		N2-32		277.8	4.63	8.1	–	–
		GPU2		155.2	2.59	19	1.8	− 134.5
		GPU4		110.9	1.85	27.1	2.5	− 235
		GPU8		61.4	1.02	30.1	4.5	− 271
DGX		GPU2		113.71	1.9	–	–	–
		GPU4		70.41	1.18	–	–	–
		GPU8		49.5	0.83	–	–	–
AWS	Somatic	C6i.8xlarge	Muse	425.1	7.09	9.6	–	–
		GPU2		65.17	1.09	13.29	6.52	− 37.97
		GPU4		61.35	1.02	12.52	6.93	− 29.88
		GPU8		22.27	0.37	11.59	19.09	− 20.23
GCP		N2_32		621.8	10.36	18.1	–	–
		GPU2		44.2	0.74	5.4	14.1	70.1
		GPU4		32.4	0.54	7.9	19.2	56.2
		GPU8		28.5	0.48	14	21.8	22.9
DGX		GPU2		36	0.6	–	–	–
		GPU4		23.84	0.4	–	–	–
		GPU8		22.7	0.38	–	–	–
AWS	Somatic	C6i.8xlarge	Mutect2	414.51	6.91	9.40	–	_
		GPU2		28.4	0.47	5.79	14.60	38.34
		GPU4		21.54	0.36	4.39	19.24	53.23
		GPU8		28.6	0.48	14.88	14.50	− 58.36
GCP		N2_32		487.7	8.13	14.2	–	–
		GPU2		32.9	0.55	4.03	14.8	71.63
		GPU4		16.7	0.28	4.1	29.3	71.29
		GPU8		31	0.52	15.2	15.7	− 7.06
DGX		GPU2		19.17	0.32	–	–	–
		GPU4		17.2	0.29	–	–	–
		GPU8		23.4	0.39	–	–	–
AWS	Somatic	C6i.8xlarge	SomaticSniper	391.9	6.53	8.88	–	–
		GPU2		83.7	1.4	17.07	4.68	− 92.28
		GPU4		134.12	2.24	27.36	2.92	− 208.11
		GPU8		144.48	2.41	75.17	2.71	− 746.54
GCP		N2_32		482.8	8.05	14.1	–	–
		GPU2		84.8	1.41	10.4	5.7	26.18
		GPU4		69.1	1.15	16.9	7	− 20.33
		GPU8		100.5	1.68	49.3	4.8	− 250.2
DGX		GPU2		77.54	1.29	–	–	–
		GPU4		65	1.08	–	–	–
		GPU8		63.5	1.06	–	–	–

AWS results presented here are for the p3 family with the NVIDIA Tesla V100 GPU, results for the p4 family with the A100 GPU are shown in Additional file 1: Table S1

### GPU performance across cloud platforms

**Fig. 2:**
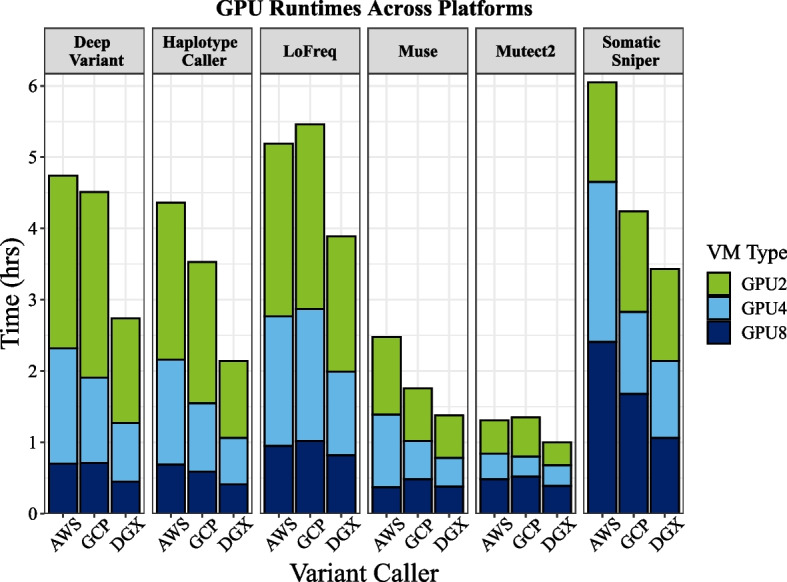
GPU benchmarking results for NVIDIA Tesla GPUs. On GCP and the DGX results are shown for A100 GPUs, whereas AWS results are shown for the V100 GPU runs

For germline callers, 8-GPU runtimes were below 43 min for HaplotypeCaller and DeepVariant across both cloud platforms. On AWS, we observed faster runtimes for the A100 compared with the V100 GPU machines (p4 vs p3 machine families), but the differences with 8 GPUs, where the number of CPUs were equal, were small for most workflows. Further, comparisons between the 2 and 4 A100 GPU machines on GCP/AWS were not precise because we were unable to limit the number of CPUs available for all AWS workflows due to constraints on available machine configurations (2 and 4 GPU machines were not available). As such, execution time differences between the two cloud platforms were biased towards AWS for some algorithms (DeepVariant and LoFreq with 2 GPUs) that were able to take advantage of the additional CPUs and memory of the larger GPU machine (see “Materials and methods” section). Although the two germline workflows scaled linearly with the number of GPUs (Fig. 2), somatic callers ran faster with 4 versus 8 GPUs for Muse on AWS (but not GCP), and for Mutect2 and SomaticSniper on both platforms (Fig. 2; Additional file 1: Figure S1). Compared with the CPU baselines, GPU runs on AWS (p4 machines with A100 GPUs) led to acceleration of HaplotypeCaller up to 65.1x, DeepVariant up to 30.7x, Mutect2 up to 56.8x, SomaticSniper up to 7.7x, Muse up to 18.9x, and Lofreq up to 3.7x (Table 1). On GCP, GPUs resulted in acceleration of HaplotypeCaller up to 65.8x, DeepVariant up to 26.5x, Mutect2 up to 29.3x, SomaticSniper up to 7.0x, Muse up to 21.8x, and LoFreq up to 4.5x.

**Fig. 3:**
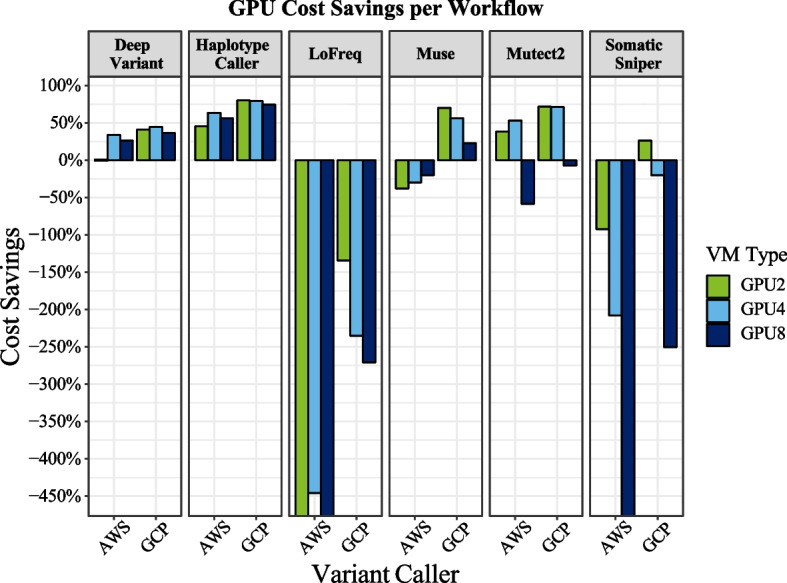
Comparison of AWS (V100 GPU machine) versus GCP GPU cost savings per variant caller. Percentage of total cost savings shows higher cost savings using GPUs in algorithms optimized for GPU-acceleration, but losses when algorithms are not well optimized

Although GPU machines are much more expensive on a per hourly basis than CPU machines, the accelerated runtimes resulted in cost savings for most algorithms (Fig. 3). Leveraging GPUs on AWS with the p3 machine (with V100 GPUs) resulted in cost savings up to 63% for HaplotypeCaller with 8 GPUs and up to 21% for DeepVariant with 8 GPUs (Additional file 1: Table S1). Using the p4 machine with the A100 GPU resulted in savings of 63% for HaplotypeCaller with 4 GPUs, 34% for DeepVariant with 4 GPUs, and 53% for Mutect2 with 4 GPUs (Table 1).

On GCP GPU runs resulted in cost savings of up to 80% for HaplotypeCaller with 2 GPUs, 44% for DeepVariant with 4 GPUs, 72% for Mutect2 with 4 GPUs, 26% for SomaticSniper with 2 GPUs, and up to 70.1% for Muse with 2 GPUs. However, on both platforms, algorithms that were not well optimized cost much more to run with GPUs rather than CPUs because the difference in runtimes was not sufficient to offset the extra GPU cost (Fig. 3; Additional file 1: Figure S4). For example, CPU runs of LoFreq cost less than $9/sample to run on both platforms, but as much as $30 with GPUs (Additional file 1: Fig. S2). Likewise, CPU runs of SomaticSniper cost less than $14.5/sample on both platforms, but as much as $75 on AWS with 8 GPUs.

For well optimized algorithms, results varied between variant callers on which numbers of GPUs were the fastest (ranging from 2 to 8); subsequently cost savings reflect a balance between speed and cost of a particular machine type that is not consistent between algorithms or cloud providers. For example, A100 GPU runs were expensive on AWS because the p4d.24xlarge machine type on-demand price is $32.8/h, whereas the A100 machine type ranges from $12.24/h for a 4 GPU machine, to $24.5/h for an 8 GPU machine. On GCP, the a2-highgpu machine types range from $7.4/h (2 GPUs) to $29.4.00/h (8 GPUs). Alternatively, CPU runs were slightly cheaper on AWS with an on-demand price of $1.36/h compared with $1.75 on GCP. Interestingly, because the somatic callers did not scale with additional GPUs, the greatest increase in acceleration (and thus cost savings) was observed with 2 GPUs. Adding additional GPUs to the somatic runs resulted in minor improvements in runtimes (if any), but substantial increases in costs/hour. Prices here are given for the northern Virginia region calculated (at the time of writing) using the pricing calculators from the respective cloud service providers. As time goes on, these machine types will likely become less expensive.

### GPU performance on the DGX

Germline workflows ran considerably faster on the DGX than on the cloud platforms, with HaplotypeCaller finishing in 24.4 min and DeepVariant finishing in 27.1 min with 8 GPUs (Fig. 2; Additional file 1: Figure S1). Somatic variant callers were not faster in most cases than the cloud platforms, and in one case, ran slower than on the cloud (SomaticSniper; Fig. 2; Additional file 1: Figure S1). Interestingly, the pattern we observed in the cloud where the 4 GPU runtimes were the fastest for Muse and SomaticSniper did not manifest on the DGX, where the 8 GPU runs were the fastest for all algorithms except Mutect2 (Fig. 2; Additional file 1: Figure S1). For Mutect2, the 4 GPU run was still the fastest on the DGX, but the 8 GPU run was faster on the DGX than on both AWS/GCP (Additional file 1: Fig. S1).

We also tested the effect of CPU number on performance of GPU runs. On AWS and GCP the GPU machine types are preconfigured (and thus unalterable) with 12 CPUs/1 GPU, but on the DGX we were able to modify the number of CPUs for each run. We found that adding CPUs does decrease runtimes (increase performance), but that reduction of runtimes plateaued after 48 CPUs (Additional file 1: Fig. S5).

## Discussion

The acceleration provided by GPU-accelerated algorithms confers several advantages to researchers. First, GPU-acceleration enables researchers to rapidly run multiple algorithms for the cost of running a single CPU run [19]. Different variant callers exhibit biases leading to slightly different variant calls [3]. Combining calls across algorithms can improve accuracy, albeit with a slightly higher type I error. Future studies could help better understand this trade off by comparing false positive and negative rates for different strategies of combining calls across algorithms such as majority rule versus consensus site calls. Another advantage of GPU-accelerated genomic workflows is that they allow researchers to process more samples on a fixed budget. Academic research programs are often constrained by limited funding; the use of GPU-acceleration may allow researchers to reduce compute costs (and labor overhead) and thus process more samples for the same amount of money. Finally, GPU-accelerated algorithms enable near-real-time decision making. Pathogen biosurveillance benefits from rapid data processing to identify novel pathogens and could help policymakers to act more quickly during an outbreak [20]. Likewise, faster clinical test processing could lead to more timely patient-care decisions in a patient-care settings.

### Cloud platform considerations

#### CPU-only runs

As more research programs migrate to cloud platforms, researchers will need to make decisions about which platform provides the most advantages for both performance and cost considerations. CPU runs were faster on the AWS c6i.8xlarge machine than on the GCP n2-32 for four algorithms, while DeepVariant and Mutect2 ran faster on GCP (Fig. 1). While the AWS machines use the 3rd generation Intel Ice Lake processors, the GCP n2 machines default to the 2nd generation Cascade Lake processors, although Ice Lake is available in some regions/zones. This difference in processor generation most likely explains the differences in runtime we observed between cloud platforms, unless unaccounted-for factors are also influencing observed variation. Past work within our research group showed that reduced runtimes driven by using the latest CPU processors outweighs the increased per-second cost (TC unpublished) suggesting that researchers should also aim to use the latest processors for CPU platforms.

Another consideration that researchers should be aware of in the near term is that AWS is migrating to newer ARM-based machine types, rather than × 86 architectures. We had trouble installing existing software on the ARM-based machines, and thus used the c6i.8xlarge machine which retains the × 86 architecture. This could present challenges for researchers in the future on AWS as the platform migrates more machine types to ARM-based architectures, necessitating the rewriting and/or compiling of common software. On GCP, we chose the N2 machine family as a balance between performance and cost. GCP does offer the compute-optimized C2 machine family, which may run faster than the N2 machines (it also uses Cascade Lake processors), but we did not benchmark those machines here. Further, future work could quantify CPU plateaus of each variant caller to help optimize the ideal CPU machine type, particularly for designing cloud-based computing clusters [13].

#### GPU considerations on the cloud

**Fig. 4:**
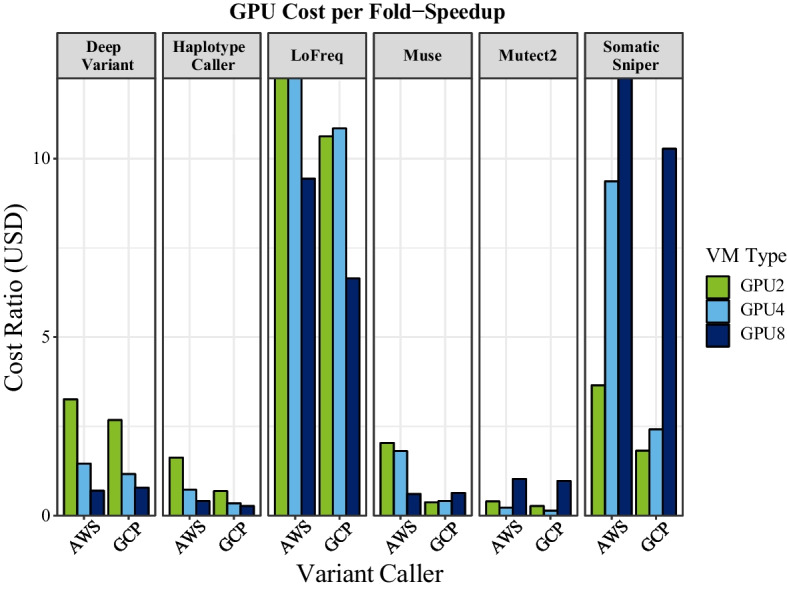
Comparison of AWS V100 versus GCP A100 GPU cost ratio per variant caller. Cost ratio is the ratio between cost per hour and fold speed-up. Cost per fold-speedup shows the benefit of harnessing GPU over CPU in select algorithms, while other algorithms are more cost-efficient with CPUs when using the version of Parabricks that we benchmarked

For germline workflows, AWS and GCP performed very similarly for both speed and cost when using 8 A100 GPUs, although the 2 and 4 GPUs runs exhibited more variation (Figs. 2, 3). To quantify the balance between cost and performance on each cloud platform, we calculated a cost ratio metric by dividing the cost of the workflow by the xSpeedup for a GPU run when compared to the CPU run for that workflow. Thus, a lower cost ratio indicates a better value for a given GPU configuration (Table 1; Fig. 4). For the germline variant callers, the best cost ratio on both platforms used 8 GPUs, and the ratio for AWS and GCP was similar enough that we feel it should not impact the choice between cloud providers. For somatic variant workflows, the best cost ratio was usually 2–4 GPUs, as these workflows were less optimized (substantially more expensive relative to speed gains) to use 8 GPUs on the cloud. Further, because LoFreq and SomaticSniper were less accelerated, their high cost ratio suggests that it is not worth the extra cost to run these workflows using GPUs with the version of Parabricks we tested. One caveat to these findings is that we used synthetic somatic data (though based on sites from a real patient), and some of our findings could be artifacts of our somatic variant sampling design. Future work could repeat similar analyses using a variety of somatic variant samples and test if different variant numbers or allele frequency variation impact algorithmic performance on GPU platforms. The newest version of Parabricks may also address some of these biases. Further, we observed faster runtimes with 4 GPUs compared with 8 GPUs for Mutect2 (on all platforms) and SomaticSniper (on GCP), and in fact, for Mutect2, using 8 GPUs was barely faster than using only 2 GPUs. We attempt to explain these results but hypothesizing that either (1) the algorithm is hard coded to only use up to 4 GPUs, or (2) the MPI used is overloaded by adding additional GPUs. We struggled to compare our results with those of [16] because the NVIDIA study only presented results for the T4 GPU machine with 4 GPUs. Further, they benchmarked on 50 × whole genome samples compared with our 30 × data, making it difficult to directly compare run times. Nonetheless, future releases of Parabricks may resolve the issue with 4 versus 8 GPUs, but more work is needed to understand the underlying causes of these patterns.

**Fig. 5:**
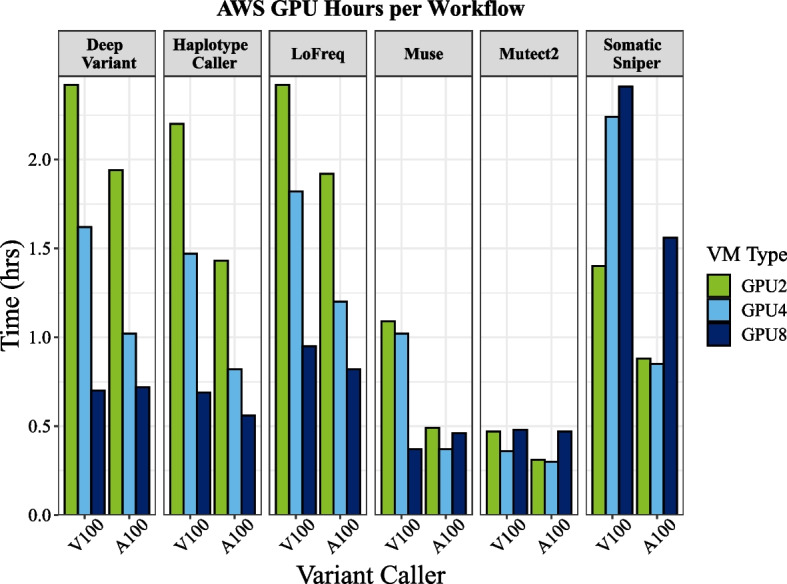
Comparison of runtimes between V100 and A100 GPU machines on AWS

GPU-accelerated bioinformatic workflows are still relatively new to the cloud, and as such, not all tools are readily available everywhere. For example, while we were conducting our analyses, Parabricks did not offer a Marketplace solution for GCP, although it has since been released. Likewise, the Marketplace solution on AWS offered a user-friendly way to access the Parabricks software suite without purchasing an annual license, but this machine image did not support the p4 machine family with the A100 GPUs. Nonetheless, although we were able to install Parabricks on the A100 machine on AWS, this machine type was not readily available (at the time of writing) in most regions, and it was difficult to procure this machine type to conduct our benchmarking. Perhaps using spot instances would have been a better solution for these difficult to procure machine types. After we conducted our study, NVIDIA has now made Parabricks free to download, and also made it available on several platforms, including Terra and Amazon Omics. Finally, we observed some decreases in runtime between the A100 and V100 GPU machines on AWS (Fig. 5). However, differences were relatively minor when using 8 GPUs—less than a minute for DeepVariant and 8 min for HaplotypeCaller. The 8 GPU p3 machine also uses newer Intel CPU processors, which may explain some of this difference. Future work could investigate the relative impact of the GPUs versus CPUs when running GPU-accelerated algorithms to better inform machine selection. Nonetheless, while the A100 machine type is difficult to obtain and was not available with the Marketplace machine image, we recommend using the V100 GPU machine without significant cost to performance (Table 1, Additional file 1: Table S1; Fig. S3).

#### On-premises computing clusters

For a myriad of reasons, some bioinformatic analysis will not migrate to the cloud, thus requiring on-premises infrastructure. Although not every institution will have a DGX cluster with A100 GPUs available, we show here that Parabricks runs well in an on-premises environment. For those looking to achieve the fastest possible runtimes in a production environment, the DGX ran considerably faster than AWS or GCP for germline callers, reducing runtimes for HaplotypeCaller by 8 min and DeepVariant by 15 min, differences that could be significant at large enough scales. We attribute these differences to the network communication between GPUs and CPUs on the machines, which is better optimized on the DGX compared with cloud-based instances, where GPUs may not be located in as close of proximity.

## Conclusions

We found that germline variant callers were well optimized with Parabricks and that GPU-accelerated workflows can result in substantial savings of both time and costs. Alternatively, somatic callers were accelerated, but exhibited substantial variation between algorithms, number of GPUs, and computing platform, suggesting that benchmarking algorithms with a reduced dataset is important before scaling up to an entire study or running at production scale. Though early days for GPU-accelerated bioinformatic pipelines, ever faster computing processors bring us closer to important societal aims such as tracking pathogens in near real-time to monitor emerging pandemics or enabling milestones in the field of personalized medicine.

## Materials and methods

### Sampling and algorithms

We benchmarked six variant callers for CPU and GPU speed and cost. Herein, we defined algorithms that are well optimized for GPUs as those that resulted in both time and cost savings when run with GPUs compared with CPU-only runs. We conducted all benchmarking on the individual ‘HG002’ from the Genome in a Bottle Consortium [21, 22] hosted by the National Institute of Standards and Technology and made available as part of the Precision FDA Truth Challenge V2 (https://precision.fda.gov/challenges/10). We down-sampled the fastq files to 30 × coverage using Samtools v1.9 [23]. We used Grch38 as our reference genome downloaded from the GATK Reference Bundle. Our germline variant calling pipeline evaluated two germline variant callers: HaplotypeCaller v4.2.0.0 [24, 25] and DeepVariant v.1.1.0 [24]. GPU benchmarking used Parabricks v. 3.7.0-1. For germline callers we used ‘Germline Pipeline’ for GATK HaplotypeCaller, and for DeepVariant we used ‘DeepVariant Germline Pipeline`. Each of these pipelines take fastq files as inputs and output unfiltered variant call format (VCF) files. CPU benchmarking was conducted by writing custom workflows using Snakemake v.6.6.1 [26], following best practices for each tool and exactly matching the workflows used by Parabricks (Data and Materials). In short, our HaplotypeCaller pipeline mapped to reference using ‘bwa mem v.0.7.15’ [27] with the ‘threads’ flag = $CPUs, sorted using Samtools [23], marked duplicates and base quality score recalibration using GATK v.4.2.0.0, and then called variants with HaplotypeCaller (-native-pair-hmm-threads = $CPUs). Likewise, our DeepVariant pipeline mapped to reference with bwa mem, sorted with Samtools, marked duplicates with GATK, then ran DeepVariant as a shell script (-num_shards = $CPUs).

Our somatic variant calling pipeline evaluated four somatic variant callers: Mutect2 [25], SomaticSniper [28], Muse [29], and LoFreq [30]. While our full workflows are described in detail on our GitHub repository, we outline the general steps for each algorithm here. Mutect2 v.4.2.0.0 was run with a single command (with –native-pair-hmm-threads = $CPUs). SomaticSniper v.1.0.5.0 was run with ‘bam-somaticsniper’ command with single threading, followed by filtering with the Perl scripts distributed with the main program. LoFreq v.2.1 was run with a single command with threads = $CPUs, and finally Muse v.2.0 was run with two steps, sump and call with single threading.

We generated synthetic somatic tumor data using SomatoSim v1.0.0 [31]. We added 198 single nucleotide polymorphisms (SNPs) at random variant allele frequencies ranging from 0.001 to 0.4 (randomly generated using custom python scripts). Sites were selected from the ICGC Data Portal ovarian cancer patient DO32536 (https://dcc.icgc.org/donors/DO32536?mutations=%7B%22size%22:50,%22from%22:151%7D). We used the BAM file from the HaplotypeCaller pipeline (i.e., MarkDuplicates, BaseRecalibration, and ApplyBQSR were run prior to the mutation process) as the input for SomatoSim. For somatic variant callers, we used the Parabricks variant caller scripts (‘mutectcaller’, ‘somaticsniper_workflow’, ‘muse’, ‘lofreq’) which take BAM files as input and output VCF files. Each Parabricks tool was compared to a compatible CPU command as listed in the Parabricks 3.7 documentation. We used Snakemake scripts as described for germline callers. For benchmarking of MuSE, we used version v2.0 and set the number of threads to 1 to replicate MuSE v1.0 lack of parallel computing because of version conflicts with MuSE v1 in our compute environment. We created a conda environment before running each workflow because we found that using the ‘–with conda’ flag in Snakemake dramatically increased run times. After initial algorithmic exploration we recorded the time of our final workflow run. We observed very minor variation in run times for serially run GPU workflows. Complete workflows are described along with all scripts necessary to repeat our analyses at https://github.com/kyleoconnell/gpu-acclerated-genomics.

### GCP configuration

Benchmarking on GCP leveraged virtual machines that were launched programmatically for CPU machines, or manually for GPU machines. On GCP, a vCPU is implemented as a single hardware hyper-thread. By default, GCP physical hardware cores use simultaneous multithreading such that two vCPUs are assigned to each core. Our CPU workflows used the ‘n2-standard-32’ machine type with Intel Xeon Cascade Lake processors with 32 vCPUs and 128 GB of memory. We assigned 1 TB of EBS storage to our instance. We launched these machines using a startup script that installed the conda environment, then ran the Snakemake workflows. All data was already loaded on a machine image, and runtimes were concatenated from each Snakemake rule using a custom script available in our GitHub repository. We also benchmarked the older generation E2 family of processors but found the run times to be much slower and thus only present the results for N2 processors here.

GPU benchmarking on GCP used the accelerator-optimized a2-highgpu machine types with two A100 GPUs, 24 vCPUs (Intel Xeon Cascade Lake processors) and 170 GB RAM, four A100 GPUs with 48 vCPUs and 340 GB RAM, and eight A100 GPUs with 96 vCPUs and 680 GB RAM. One virtual machine was utilized with 4 TB of elastic block storage, which we stopped and resized between runs.

### AWS configuration

Benchmarking on AWS also used multiple virtual machines for CPU and GPU benchmarking. Similar to GCP, AWS assigns two vCPUs to each physical core to enable multi-threading. CPU benchmarking used the C6i.8xlarge machine type, which has a 3rd generation Intel Xeon Scalable processor (Ice Lake 8375 C) with 32 vCPUs and 64 GiB RAM. We assigned 800 GB of EBS storage to our instance. We did some preliminary testing with the new ARM-based processors (C7g family) but had issues with installing several of the dependencies (particularly with mamba/conda), suggesting that a migration to ARM-based processors may prove problematic for bioinformatics in the cloud.

We benchmarked two GPU machine families. First, we benchmarked the p4 machine family which is similar to GCP a2-highgpu machines utilizing the latest NVIDIA A100 Tensor Core GPUs with 8 GPUs with 96 vCPUs (Intel Xeon Cascade Lake P-8275CL) and 1152 GiB RAM. AWS currently only has one machine type with A100 GPUs, the p4d.24xlarge, which only runs with 8 GPUs. To ensure consistency with GCP, we ran the 8 GPU machine, but specified the number of GPUs to use in our Parabricks commands for the smaller numbers of GPU runs. As this machine type was not compatible with the marketplace image (see below) we installed Parabricks manually using scripts provided by NVIDIA. When possible (–cpu flag available) we limited the number of CPUs available with the p4 machine, but most analysis did not allow us to control the number of CPUs. For example, we ran HaplotypeCaller with 2 GPUs, but 96 CPUs, compared with on GCP where the machine had 2 GPUs and 24 CPUs.

To compare GPU and CPU configurations directly with GCP, we further benchmarked the p3 machine family using the ‘NVIDIA Clara Parabricks Pipelines’ AWS Marketplace image. At the time of writing the image supported V100 GPUs (but not A100 GPUs), which are an older model of Tensor Core GPU, on machine types p3.8xlarge with 4 GPUs, and 32 Intel Broadwell E5-2686 v4 CPUs. We also benchmarked on the p3dn.24xlarge with 8 GPUs and 96 Intel Skylake 8175 CPUs. The Marketplace image also had Parabricks preinstalled at a cost of $0.30/h (NVIDIA has since made Parabricks free). This configuration allowed us to directly compare 4 and 8 GPU machines with equal CPU numbers between AWS and GCP.

### DGX configuration

We also conducted GPU benchmarking on an NVIDIA DGX Cluster (DGX SuperPOD), which is a computing cluster with six DGX A100s, each of which contains eight NVIDIA A100 GPUs and 64 core AMD Rome CPUs with 1 TB RAM. Although the DGX cluster is composed of four DGX A100 components, resulting in a total of 48 A100 GPUs available, Parabricks is only able to run on a single DGX A100 system, thus limiting any Parabricks analyses to 8 GPUs. Jobs were launched using a Kubernetes-based scheduler, allocating a max memory of 300 GB, and matching the GPU and CPU configurations of the GCP/AWS runs, except GATK HaplotypeCaller. For this workflow, we benchmarked times for 8 GPUs using 24, 48, 96, and 124 CPUs to test the effect of the number of CPUs on execution time. For all other algorithms, we ran at least three iterations of each run to ensure consistency of results, and present the time of the final run.

## Supplementary Information

**Additional file 1**. Additional results of benchmarking on AWS. Table S1 shows NVIDIA A100 GPU machine benchmarking results, and figures show benchmarking the NVIDIA V100 GPU machine.

## Data Availability

The datasets supporting the conclusions of this article are available in the GitHub repository accessible at https://github.com/kyleoconnell/gpu-acclerated-genomics.
